# CPA-7 influences immune profile and elicits anti-prostate cancer effects by inhibiting activated STAT3

**DOI:** 10.1186/s12885-016-2488-6

**Published:** 2016-07-19

**Authors:** Meihua Liang, Fei Zhan, Juan Zhao, Qi Li, Jiazi Wuyang, Guannan Mu, Dianjun Li, Yanqiao Zhang, Xiaoyi Huang

**Affiliations:** Department of Endocrinology, the Second Affiliated Hospital of Harbin Medical University, Harbin, 150086 China; Department of Gastrointestinal Medical Oncology, The Affiliated Tumor Hospital of Harbin Medical University, Harbin, 150081 China; Biotherapy Center, Tumor Hospital of Harbin Medical University, Harbin, 150081 China; Center of Translational Medicine, Harbin Medical University, Harbin, 150086 China

**Keywords:** Prostate cancer, CPA-7, Regulatory T cell, STAT3, CD4^+^ T cell

## Abstract

**Background:**

Platinum-based chemotherapy is emerging as the first line of treatment for castration resistant prostate cancer. Among the family of platinum (IV)-based compounds, a member known as CPA-7 inhibits the growth of multiple cancer cell lines. However, how and to what extent CPA-7 elicits its anti-prostate cancer effects in vivo is largely unknown.

**Methods:**

In this study, we firstly assessed the potential toxicity of the synthesized CPA-7 in a prostate cancer model as well as in normal mice. Next, we evaluated the in vitro effects of CPA-7 on the growth of prostate cancer cells using cell counting assay, and calculated the tumor sizes and cumulative survival rate of the tumor bearing mice by Kaplan-Meier method during CPA-7 treatment. Then we measured the expression level of the activated form of STAT3 (one targets of CPA-7) and its transcriptive activity post CPA-7 treatment by synergistically using western blot, IHC, and firefly luciferase reporter assays. Finally, effects of CPA-7 on immune cell trafficking in the tumor draining lymph nodes and in the spleens are evaluated with flow cytometry.

**Results:**

Treatment with CPA-7 significantly inhibited growth of prostate cancer cells in vitro, and also in mice resulting in a prolonged survival and a decreased recurrence rate. These therapeutic effects are due, at least in part, to functional depletion of STAT3 in prostate tumor tissue as well as in the surrounding areas of tumor cell invasion. CPA-7 treatment also resulted in a reduced level of regulatory T cells and increased levels of cytotoxic T and T helper cells in the spleen and in tumor infiltrating lymph nodes. This favorable effect on immune cell trafficking may account for the amnestic immune response against recurrent prostate cancer.

**Conclusions:**

CPA-7 is a promising new therapeutic agent for prostate cancer that both inhibits tumor cell proliferation and stimulates anti-tumor immunity. It has potential as first line treatment and/or as an adjuvant for refractory prostate cancer.

**Electronic supplementary material:**

The online version of this article (doi:10.1186/s12885-016-2488-6) contains supplementary material, which is available to authorized users.

## Background

Androgen deprivation therapy (ADT) is used as a first line therapy for hormone sensitive, metastatic prostate cancer (PC). ADT acts by depleting testosterone levels, leading to substantial cancer control and palliation lasting an average of 20–30 months [1, 2]. However, when prostate cancer has progressed to the castrate resistant stage and is no longer expected to respond to treatment with ADT, the first line of treatment is typically with a platinum compound such as carboplatin, cisplastin, oxaliplatin or satraplatin, combined with docetaxel or etoposide [3, 4]. These combined treatments have recently shown promising results, especially for neuroendocrine prostate cancers which are usually resistant to androgen deprivation therapies [4, 5]. Among the family of platinum (IV)-based compounds, a member known as CPA-7 with a molecular formula of fac-[PtCl3(NO2)(NH3)2] induces apoptosis in multiple cancer cell lines, including human breast, lung, colon and prostate cancer cells and in mouse melanoma cells [6, 7]. However, how and to what extent CPA-7 elicits its anti-prostate cancer effects in vivo is largely unknown.

Signal transducer and activator of transcription 3 (STAT3) is, with phospho-STAT3 (pSTAT3) as its activated form, an important oncogenic protein well known for its role in tumor cell proliferation, survival, and invasion. Constitutively activation of STAT3 is found in approximate 70 % of the solid malignancies, including hematological malignancies as well as diverse solid tumors such as head and neck, breast, lung, gastric, hepatocellular, colorectal and prostate cancers [8, 9]. In prostate cancer, aberrant IL-6/STAT3 signalling is one of the most involved pathways during the transition of metastatic disease [10]. In addition to directly function on tumor cells, STAT3 also plays pivotal roles in anti-cancer immunity, not only as a potent negative regulator of T helper 1 (TH1)-cell-mediated inflammation, but also as an important activator of genes that are crucial for immunosuppression [11]. Therefore, targeting STAT3 could well reduce tumorigenesis and modulate tumour-induced immunosuppression.

To date, STAT3 specific inhibitor is still under development [12]. Among the STAT3 inhibitors, such as SHP2, HJC0152, Benzyl isothiocyanate [13], CPA-7 was found efficiently targeting STAT3 [7, 14]. At low doses, CPA-7 depletes the DNA binding capacity of STAT3 leading to down-regulation of genes down-stream of STAT3 and, ultimately, to tumor regression [7]. Another study reported that functional depletion of STAT3 elicited by CPA-7 treatment resulted in down-regulation of IL-10 and IL-6, and up-regulation of IL1-β in glioma cells [15]. In addition to STAT3, report from Assi H demonstrated that CPA-7 also inhibited the activity of STAT1 and NF-kB, as evidenced by the firefly luciferase assays [16], suggesting the pleiotropic effects of CPA-7. However, STAT3 may possibly account for the most sensitive one because CPA-7 induced functional depletion of STAT3 at very low concentration. In prostate cancer, the in vivo effects of CPA-7 on STAT3 and accordingly on immune profile are still need to be clarified.

To address the questions above, in the present study, we first assessed the potential toxicity of CPA-7 in vivo. In addition, we evaluated the effects of CPA-7 on the growth of prostate cancer cells in vitro and in vivo, and its effects on immune cell trafficking. Finally, we measured the effects of CPA-7 on the expression level of the activated form of STAT3 and its transcriptive activity post CPA-7 treatment.

## Methods

### Antibodies, reagents and kits

Rabbit anti-mouse p-STAT3 antibody (Y705) was purchased from Cell Signaling (USA). HRP-conjugated goat anti-rabbit antibody and Mouse Regulatory T Cell Staining Kit were from eBioscience (USA). Rabbit anti-mouse-CD8 antibody was from BD Pharmingen (USA). ABComplex/HRP (Rabbit IgG, Cat SK-5100), AEC substrate kit (SK-4200), 3-amino-9-ethylcarbazole, and normal goat serum were from Vector Laboratories (USA). Fugene HP transfection reagent was from Roche Applied Science (USA). STAT3 Luciferase Reporter Vector was from Beyotime (China). pRL-TK vector was from Promega (USA). Firefly & Renilla Luciferase Assay Kit was from Biotium Inc (USA). All of the other reagents were from Sigma Aldrich (USA) except indicated.

### Cell line

Murine prostate cancer cell line RM-9 was a gift from Dr. Mouraviev in Duke University and was grown at 37 °C in complete Dulbecco’s Modified Eagle Medium (DMEM, Life Technologies, USA), supplemented with 10 % fetal bovine serum (Biological Industries, Israel), 2 mM L-glutamine, 50 IU/mL penicillin and 50 μg/mL streptomycin (Life Technologies, USA) and in a humidified atmosphere of 5 % CO2 and air. HEK293T cells, which were from American Type Culture Collection (ATCC, USA) and used for the luciferase reporter assay, were cultured in the same conditions as the RM-9 cells.

### CPA-7 synthesis

CPA-7 was synthesized as previously described [6]. Briefly, 300 mg of yellow sis-[PtCl2(NH3)2] (0.1 mmol) was suspended in a solution of 0.074 g KCl (0.1 mmol) in 25 ml distilled water. The mixture was protected from light by aluminum and stirred, while bubbling nitrogen dioxide through the solution at a rate of approximately one bubble per second. The cylinder of NO_2_ was warmed in a water bath at ~45 °C in order to increase the vapor pressure of the gas. When the reaction mixture was completely dissolved to form a dark turquoise solution, NO_2_ bubbling was terminated and air was bubbled through the solution overnight. The following day, if the solution remained yellow, the reaction was considered complete and the mixture was pumped with vacuum dryer to dryness. The yellow powder was collected and stored at −80 °C until use.

### Cell counting

RM-9 cells were seeded into 24-well plates at 2 × 10^4^ cells/well and incubated overnight at 37 °C. Then cells were treated daily with fresh culture medium containing either CPA-7 at concentrations of 5, 10 or 20 μM or with 1 % DMSO, the vehicle control. At each treatment, the cells in 3 parallel wells per conc of CPA-7 or DMSO were collected by trypsinization. Suspended cells from individual wells were diluted in trypan blue exclusion medium and counted with the use of a haemocytometer. Trypan blue stained cells were considered non-viable. The total number of viable cells in each group were recorded.

### Western blot

RM-9 cells were seeded into 6-well plates at 5 × 10^5^ cells/well and treated with either CPA-7 at 10 μM or 1 % DMSO vehicle for 24 h. The cells were collected by trypsinization and washed twice with cold PBS. Total protein was extracted by adding RIPA buffer to the cell and sonicating the mixture on ice. The lysate was centrifuged at 14,000 g, 4 °C for 30 min and the supernatant was collected. An amount of 40 μg of total protein was separated on a 12 % SDS-PAGE gel and transferred onto a PVDF membrane. Then the membrane was immersed in PBS containing 5 % fat-free milk for 4 h at room temperature. Primary antibody was added into the blocking buffer at 1:500 dilution overnight at 4 °C. After the membrane was washed with TBS-T buffer, secondary antibody was applied at 1: 2000 dilution in blocking buffer for another one hour. After washing with TBS-T buffer, the membrane was incubated with luminol substrate solution (Transgene, China). Signal of the target protein was sensitized and developed with an X-ray film.

### Firefly and Renilla Luciferase assay

RM-9 cells were seeded into 12-well plates one day before transfection. When cell growth was ~50 % confluent, transfections were performed. Briefly, for each well, 1 μg of pSTAT3-Luc vector plus 0.05 μg pRL-TK vector (background control) were mixed with Opti-Medium and incubated for 10 min at room temperature, followed by addition of 2.5 μl FuGene HD transfection reagent. After incubation for another 15 min, the transfection complex was added to each well and the plate returned to the incubator. Twenty-four hours post transfection, the cells were treated with culture medium containing CPA-7 at 5 μM, 10 μM or 1 % of vehicle-DMSO. After another 24 h, cells were collected and Luciferase assay was performed following the manufacturers instructions. In brief, cells were treated with lysis buffer for 15 min. After centrifugation, 10 μl of supernatant plus 50 μl of firefly luciferase assay solution was mixed together in a luminometer tube. Then the firefly luciferase activity was measured with a luminometer. Secondly, 10 μl of supernatant was added into a new tube to which was added 25 μl of Renilla Luciferase Assay Enhancer followed by 25 μl of Renilla Luciferase Assay Solution. This complex was then measured with the luminometer to determine the background fluorescence which served to normalize the transfection and the amount of protein loaded.

### Tumor model and CPA-7 treatments

**Fig. 1:**
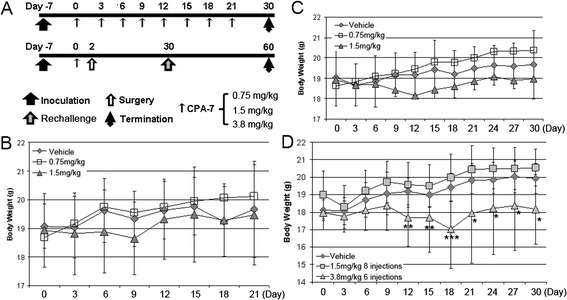
Study design and side effects of CPA-7 for in vivo treatment evaluated by body weight. **a**, schematic illustration of the study design. CPA-7 was administrated by tail vein injection at three different doses. Injection of PBS containing 1 % DMSO (vehicle) served as control. An additional three groups of animals were used to evaluate tumor recurrence. These mice were subjected to surgical ablation two days post CPA-7 treatment and followed up for total of 60 days. **b**, effects of CPA-7 treatment on body weight in RM-9 tumor bearing mice. *n* = 9 ~ 11 for each group. **c**, effects of CPA-7 treatment on body weight in tumor bearing mice after surgery. *n* = 10 ~ 12 for each group. **d**, effects of CPA-7 treatment on body weight in healthy mice. *n* = 10 for each group. *, *P* < 0.05, **, *P* < 0.01, ***, *P* < 0.001 when compared with vehicle group

C57BL/6 J mice, 6–8 weeks old, were purchased from Vital River (Beijing, China) and housed in the vivarium. All the animals were handled in accordance with the established animal care policy and all animal experiments were approved and supervised by the Harbin Medical University Institutional Animal Care & Use Committee. As indicated in the Study Design (Fig. 1a), RM-9 tumor model was established by subcutaneously injecting 8 × 10^4^ RM-9 cells into the right hind limb of each mouse. Tumors were allowed to grow for about 7 days until they reached 5 ~ 6 mm in diameter before administration of CPA-7 every three days via tail vein injection. The animals were checked for tumor development for 30 days. During this period the tumor volumes and sizes, and the body weights were recorded daily. The humane endpoints were defined by overall tumor volume exceeding 2 × 10^3^ mm^3^ or when the tumors developed ulcers.

In a parallel experiment to determine anti-tumor memory following treatment with CPA-7, two days following the first administration of CPA-7 or sham treatment, the primary tumor along with the skin above the tumor tissue was surgically removed. Animals were continuously subjected to CPA-7 treatment and regarded as completely recovered from surgery and free of recurrence when no local tumor was observed four weeks after surgery [17]. Rechallenge was performed 30 days after surgery in recurrence free animals. A total of 5 × 10^3^ RM-9 cells were subcutaneously injected into the contralateral limb of the mouse. The animals were then followed for another 30 days, during which the tumor recurrence and survival rate were recorded.

### Flow cytometry

Three and twelve days post CPA-7 treatment, three mice in each group were randomly euthanized and the spleens and tumor draining lymph nodes (TDLNs) were harvested for flow cytometry. Briefly, single cell suspensions from pooled spleens or TDLNs (1 × 10^6^/staining) were prepared and FcγR was blocked for 15 min by CD16/32 antibody (Fc Block) before cell surface staining. Following cell surface staining of CD4 and CD25, intracellular staining of Foxp3 to identify Tregs was performed with fixation/permeabilization working solution (eBioscience). The stained cells were processed with a FACS Aria II flow cytometer (BD, USA). Tregs were identified by CD4^+^Foxp3^+^ markers. Cytotoxic T lymphocytes are represented as CD8+ population in spleen and TDLN.

### Immunohistochemistry (IHC)

Three and twelve days after CPA-7 treatment, three mice from each group were sacrificed. The tumor tissues were collected and fixed overnight with 10 % formalin. After embedded in paraffin, tissues were cut at 5 μm thickness and mounted to glass slides. The tissue sections were deparaffinized, rehydrated, and subjected to antigen retrieval in an EDTA-containing buffer for 30 min at 95 °C. Endogenous peroxidase activity was blocked with 0.3 % hydrogen peroxide for 15 min at room temperature. Then the slides were incubated with 10 % normal goat serum for 30 min and then with rabbit anti-mouse phospho-STAT3 antibody (diluted with PBS at 1:50) at 4 °C overnight. Following washes with PBS, the slides were incubated with biotinylated secondary antibodies (diluted with PBS at 1:200) for 30 min at room temperature and then with avidin-biotin-horseradish peroxidase complex for another 30 min. Detection was performed using 0.125 % aminoethylcarbazole chromogen. After counterstaining with Mayer’s hematoxylin (Sigma), the slides were mounted. Under microscope, nuclei of phospho-STAT3 positive tumor cells were in purple, while nuclei of phospho-STAT3 negative cells remained in blue. Scoring was conducted by evaluating at least 10 different fields per sample and independent observations by three different pathologists.

### Statistical analysis

Comparison between the observation and the control groups was carried out mostly by using the Student’s *t*-test with the results expressed as mean ± SD. The cumulative survival rate was calculated by using the Kaplan-Meier method and difference in the survival rate was evaluated by the log-rank test using Softstat software. *P* < 0.05 was considered to be statistically significant.

## Results

### Synthesized CPA-7 had little impact on the body weight of the mice when applied at no more than 1.5 mg/kg

Because side effects are a major concern with platinum-based antineoplastics, we evaluated the safety of CPA-7. RM-9 tumor bearing mice were treated initially with two doses of CPA-7 administered by tail vein injection when the tumor size reached 5 mm in diameter. Vehicle (1 % DMSO in PBS) injected mice were used as controls. All three groups of mice were weighed every three days and each mouse given 8 separate injections. As shown in Fig. 1b, the body weights of mice treated with either 0.75 mg/kg or 1.5 mg/kg CPA-7 increased slightly with time throughout the entire period of observation. Body weight differences between vehicle control and CAP-7 treated mice were not statistically different although the mice treated with 1.5 mg/kg of CPA-7 were lighter than mice in the other two groups during most of the follow-up period. Since the ever-growing tumor mass may compensate the loss of body weight resulted from CPA-7 treatment, we next surgically removed the tumors 2 days after CPA-7 injection and continued the follow-up to the end point. As shown in Fig. 1c, mouse weights in the CPA-7 treated groups are not statistically different from those in the control group.

To rule out the impact of tumor burden and surgery on body weight, we next tested the safety of CPA-7 in healthy mice with an additional higher dose of 3.8 mg/kg, which is equivalent to 10 μM/kg. Similar to results obtained in tumor bearing mice, healthy mice subjected to 1.5 mg/kg of CPA-7 had steady increases in body weight. However, when the CPA-7 dose was increased to 3.8 mg/kg, we observed significant reductions in body weight after the 4^th^ (day 15) injection as compared with the control group. Though the weight promptly returned to the basal level 3 days after termination of CPA-7 injections, statistical significances between the group treated at 3.8 mg/kg and the control group continued over the observation from the time point of 4^th^ injection on which the differences initially occurred (Fig. 1d).

### Synthesized CPA-7 inhibits RM-9 cell growth in vivo and in vitro

**Fig. 2:**
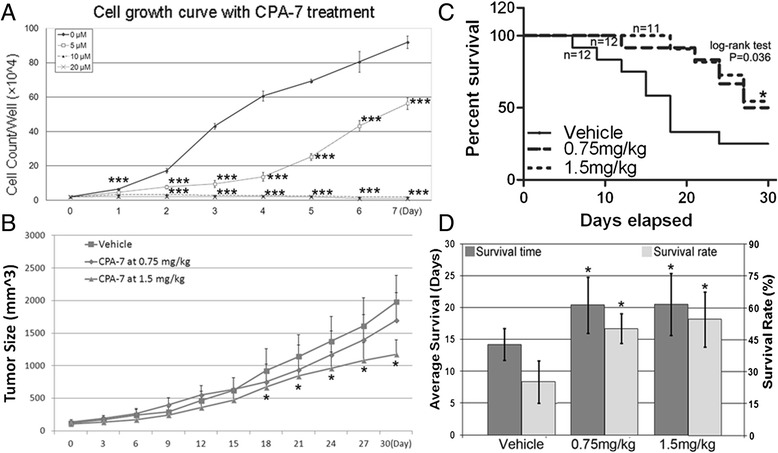
Therapeutic effects of CPA-7 on RM-9 tumor. **a**, cell counting assay using in vitro cultured RM-9 cells. ***, *P* < 0.001 when compared with the vehicle control. **b**, dynamic curve of tumor sizes during follow-up. The tumor bearing mice were monitored for 30 days, during which tumor sizes were recorded every three days. *n* = 12 ~ 15 for each group. *, *P* < 0.05 when compared with the vehicle group. **c**. Kaplan-Meier plots for overall survival. Tumor bearing mice were treated with CPA-7 at indicated doses or with vehicle and monitored for 30 days. *n* = 11 ~ 12 for each group. CPA-7 treatment at 1.5 mg/kg significantly prolonged the overall survival of the animals when compared with the vehicle group (*, log-rank *P* = 0.036). CPA-7 treatment at 0.75 mg/kg also had a trend to prolong the overall survival of the tumor bearing mice (log-rank *P* = 0.051). **d**, impact of CPA-7 on median survival time and survival rate at the end of follow-up. Both median survival time (left Y axis) and survival rate (right Y axis) were calculated in the end of the follow-up and plotted to different dose of CPA-7. Each group had 11 ~ 12 mice. *, *P* < 0.05 when compared with the vehicle group

In order to determine to what extent our synthesized CPA-7 is capable of inhibiting tumor growth, RM-9 cells were treated with CPA-7 at various concentrations, and the vehicle DMSO was used as control. As seen in Fig. 2a, the number of RM-9 cells treated with 5 or 10 μM CPA-7 decreased over time whereas the number of DMSO-treated control cells increased significantly (*P* < 0.001) beginning with day 2 post-treatment. CPA-7 at 10 μM had a stronger tumor inhibitory effect than at 5 μM. Seven days after treatment, the number of the cells in cultures treated with 10 μM CPA-7 was approximately the same as the starting number, compared with nearly half the number of the primary cells in cultures treated with 20 μM of CPA-7. Overall, it is apparent that CPA-7 strikingly inhibits proliferation of RM-9 cells.

We next evaluated the ability of CPA-7 to inhibit the growth of RM-9 tumors in vivo. As seen in Fig. 2b, on Day 0, on which was the initial treatment of the mice with either CPA-7 or the vehicle control, the tumor sizes were similar in all groups. Thereafter, tumors in mice treated with CPA-7 were smaller than those in vehicle control animals. CPA-7 at 1.5 mg/kg elicited the strongest tumor inhibitory effect, followed by CPA-7 at 0.75 mg/kg. After 18 days, which was the 7th treatment cycle, RM-9 tumors in mice treated with 1.5 mg/kg CPA-7 (1167 ± 228 mm^3^) were found to be significantly (*P* < 0.05) inhibited in size relative to the control group (1958 ± 375 mm^3^) whereas those in mice treated with 0.75 mg/kg CPA-7 (1710 ± 417 mm^3^) were not statistically different from the control group (*P* ≥ 0.05). Log-rank analysis demonstrated that during the observation period, tumor bearing mice subjected to 1.5 mg/kg CPA-7 (*P* = 0.036), but not 0.75 mg/kg, had significantly higher survival time than vehicle controls, though dose of 0.75 mg/kg showed a trend to increase the survival of the tumor model (Fig. 2c). At the end of the follow-up, both doses led to significantly higher median survival time and survival rate when compared with the control group (Fig. 2d), and the high concentration of CPA-7 led to higher survival rate than low concentration, while no statistical significance was found between the two CPA-7 treated groups (Fig. 2d). To determine the effects of CPA-7 on tumor recurrence, primary tumors were surgically removed two days after the starting point of CPA-7 treatment. Thirty days post surgery, the animals were re-challenged with 5000 RM-9 cells administered sub-q into the contralateral limb and followed for another 30 days. At the end of the observation period, mice treated with both concentrations of CPA-7 were found to have significantly (*P* < 0.05) lower recurrence rates (6 out of 12 in the 0.75 mg/kg CPA-7 treatment group and 4 out of 12 in the 1.5 mg/kg CPA-7 group) when compared with the vehicle control group (9 out of 12).

### CPA-7 down-regulates the activity of STAT3 in vivo and in vitro

**Fig. 3:**
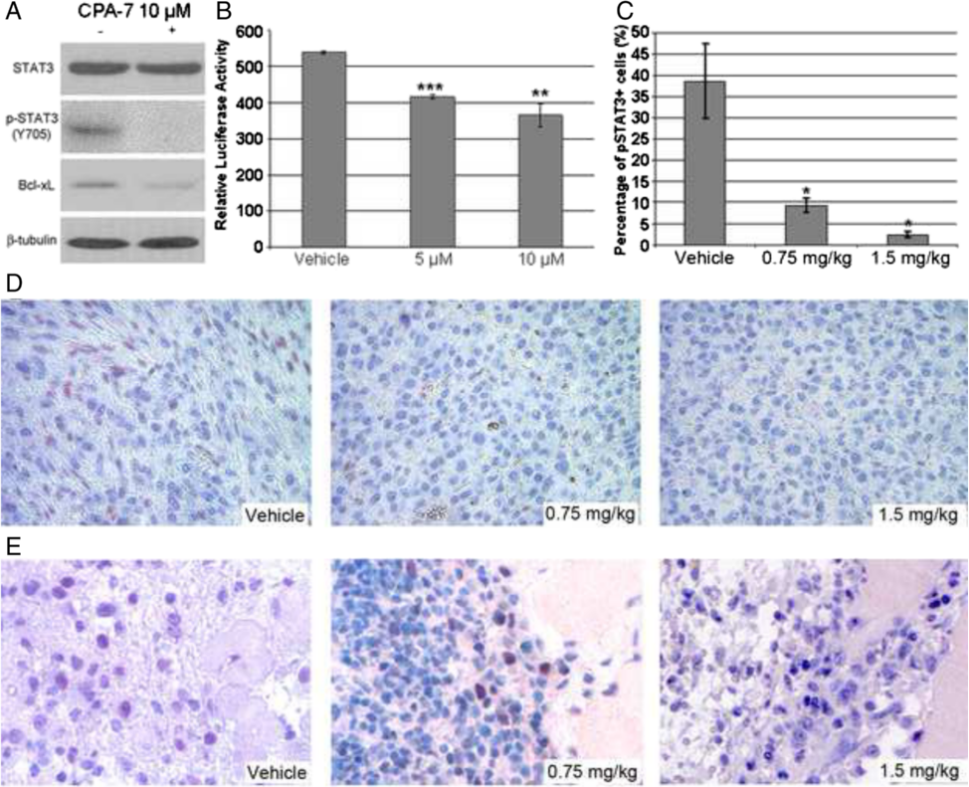
Inhibitory efficacy of CPA-7 on STAT3 activity. **a**, functional depletion of activated STAT3 in RM-9 cells. RM-9 cells were incubated for 24 h with CPA-7 at the indicated doses or vehicle containing 1 % DMSO, respectively. Total protein was isolated and thereafter western blot was performed against STAT3, pSTAT3, and Bcl-xL. Immunobloting against β-tubulin was included as control. **b**, transcriptive activity of STAT3 determined by luciferase reporter assay. HEK-293 T cells were maintained and transfected with vectors as indicated in the Materials and Methods. After CPA-7 or vehicle treatment, total protein was extracted and STAT3 specific firefly luciferase activity was determined, with normalization to renilla luciferase activity. **c**, **d** and **e**, in vivo level of pSTAT3 after CPA-7 treatment. pSTAT3 positive cells were calculated by individually counting three different random microscope fields in the central regions of the tumor tissues (**d**) or in the infiltrating areas (**e**). Percentage of positive rate in the central region of the tumor tissues was obtained by dividing each number with the total number of cells in the corresponding field (**c**). *, *P* < 0.05, **, *P* < 0.01, ***, *P* < 0.001 when compared with the vehicle group

We previously reported the content of endogenously expressed activated form of signal transducer and activator of transcription 3 (phospho-STAT3, p-STAT3) in prostate cancer cells [18]. In the present study, we found that CPA-7 depletes p-STAT3 in cultured RM-9 cells as revealed by Western blot analysis (Fig. 3a). Expression of p-STAT3 was effectively depleted by 10 μM CPA-7 as indicated when comparing the second lane with the first lane in the second uppermost panel. In addition, as shown in the uppermost panel, total STAT3 level in RM-9 cells changed only slightly post CPA-7 treatment. These results indicate the CPA-7 doesn’t affect the expression of total STAT3, but deregulates its activity. The significant down-regulation of Bcl-xL, one well documented target of pSTAT3, further verified the functional depletion of STAT3 after CPA-7 treatment. To further determine the effect of CPA-7 on STAT3 transcriptional activity, luciferase assays were performed using pGAS-Luc transfected HEK293 cells expressing a luciferase gene construct under control of a Stat3-specific promoter. HEK293 cells were treated with CPA-7 at 5 and 10 μM for 24 h after which their total proteins were extracted and luciferase activity was measured. As shown in Fig. 3b, treatment with 10 μM CPA-7 caused approximately a 32 % reduction in STAT3 activity when compared with the negative control, which represented the endogenous STAT3 activity. Treatment with 5 μM CPA-7 led to a 23 % reduction in STAT3 activity.

Next, we determined whether CPA-7 inhibits STAT3 activity in the RM-9 tumor model, CPA-7 was injected in the tail vein at final concentrations of either 0.75 or 1.5 mg/kg. Three days after administration, primary tumors were removed and p-STAT3 measured by immunohistochemistry (IHC) [18]. As shown in Fig. 3c and d, it is apparent that CPA-7 treatment can significantly (*P* < 0.01) reduce the level of pSTAT3 in RM-9 tumor tissue in a dose dependent manner, where CPA-7 treatment at even the low dose of 0.75 mg/kg led to more than a 3.6-fold decrease in the pSTAT3 level (Fig. 3c, *P* = 0.005). Likewise, pSTAT3 level was strikingly down-regulated on Day 12 during the CPA-7 treatment (Additional file 1: Figure S1). In addition to the central region of tumor tissues, sections prepared from the tumor boundary were also subjected to IHC towards pSTAT3. As anticipated, before CPA-7 treatment, the level of p-STAT3 was high in the infiltrating area between the tumor tissues and the adjacent muscle tissues. Statistically significant inhibition (*P* < 0.05) of pSTAT3 was found after CPA-7 treatment when compared to the vehicle control (Fig. 3e). The inhibiting indexes in tumor boundary were compatible with the inner tumor tissue. These results clearly indicate that CPA-7 inhibits the pSTAT3 level in vivo.

### CPA-7 treatment improves the anti-tumor immune response in mice

**Fig. 4:**
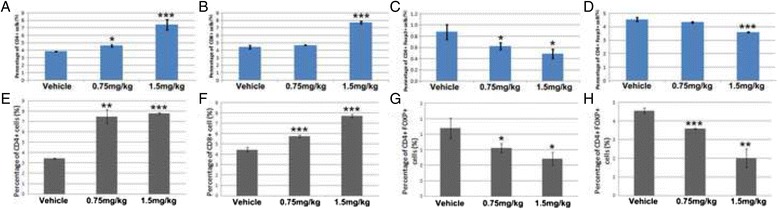
CPA-7 effects on immune profile. CD4+ (**a**), CD8+ (**b**), and Treg (**c**) in spleens and Treg in tumor draining lymph nodes (TDLNs) (**d**) were determined three days after CPA-7 treatment at indicated doses. Likewise, CD4+ (**e**), CD8+ (**f**), and Treg (**g**) in spleens and Treg in TDLNs (**h**) were determined 12 days after CPA-7 treatment at indicated doses. *n* = 3 for each group. *, *P* < 0.05, **, *P* < 0.01, ***, *P* < 0.001 when compared with the vehicle group

Regulatory T cells (Tregs) account for a small fraction of CD4+ T cells but inhibit immune responses and have been implicated in immune tolerance to tumors [19]. In contrast, CD8+ cytotoxic lymphocytes recognize and eradiate tumor cells [20]. To investigate whether CPA-7 treatment at dose of 0.75 mg/kg or 1.5 mg/kg affects T cell subsets, single lymphocyte suspensions were prepared from spleens and tumor infiltrating lymph nodes (TDLNs) at 3 and 12 days after the first treatment of CPA-7. The cell suspensions were stained using anti-CD8, anti-CD4 and anti-FOXP3 antibodies and analyzed by flow cytometry. Three days after CPA-7 treatment with either dose, the number of CD4+ cells in the spleen increased more than 1.9 fold when compared with the vehicle control (Fig. 4a, *p* < 0.05 for CPA-7 at 0.75 mg/kg and *p* < 0.001 for CPA-7 at 1.5 mg/kg). In addition, the number of CD8+ cells in the spleen increased about 1.7 folds in animals treated with 1.5 mg/kg CPA-7 (Fig. 4b, *p* < 0.001), while the low-dose of CPA-7 only marginally changed the number of CD8+ cells. In contrast, the number of splenic Treg cells was significantly decreased post treatment with CPA-7 at either dose (Fig. 4c; 33 % reduction for CPA-7 at 0.75 mg/kg, *p* < 0.05; and a 44 % reduction for CPA-7 at 1.5 mg/kg, *p* < 0.05). For the TDLNs treatment with 0.75 mg/kg CPA-7 resulted in a non-significant decrease in Treg cells whereas treatment with 1.5 mg/kg resulted in a significant 20 % decrease in Treg cells (Fig. 4d, *p* < 0.001). Similar but more consistent changes in the immune profile were found at Day 12 during the course of CPA-7 treatment (Fig. 4e-h). These results suggests that after CPA-7 treatment there is a long-lasting increase in cytotoxic T cells and a decrease of Tregs in spleen as well as in TDLNs after CPA-7 treatment.

## Discussion

The progression of prostate cancer to castration resistance results in a significant shortening of the anticipated survival period and limited treatment regimens. In spite of the significant progress in anticancer drug development for patients with metastatic castration-resistant prostate cancer in the past few decades, conventional chemotherapies are still widely used for prostate cancer worldwide [21, 22]. However, clinical application of conventional chemotherapeutic drugs including platinum-based chemicals is significantly thwarted by numerous undesirable side effects such as severe kidney problems, allergic reactions, hemorrhage, and hearing loss [23]. Recently, chemicals that possess the potential of modulating anti-tumor immunity have gained significant attention for cancer treatment [22, 24, 25]. For example, Low-dose decitabine treatment recruit NK and CD8+ T cells, promotes their production of IFNγ and TNFα, and extends the survival of ovarian cancer [15]. Gemcitabine combined with cisplatin chemotherapy significantly reduce the population of CD4 + CD25 + FOXP3+ Treg in patients with non-small cell lung cancer [8]. However, to date there is no direct evidence demonstrating the modulator roles of the platinum-based chemical, CPA-7, on adaptive immunity.

To develop a feasible strategy for treatment of prostate cancer with CPA-7, we initially evaluated its potential toxicity in mice when administered at different concentration. Assi et al. tested the potential toxicity of CPA-7 in a melanoma model [16]. They found there’s no obvious toxic effect on liver and spleen when CPA-7 was administrated at 5 mg/kg. In our study, we used body weight as evaluating index to find CPA-7 is safe to body weight when the dose of a single injection is less than 1.5 mg/kg for long term administration. It is of note that treatment with CPA-7 at 1.5 mg/kg significantly inhibited growth of subcutaneously established prostate tumors, but treatment with 0.75 mg/kg was ineffective. Both doses of CPA-7 significantly prolonged both the survival time and rate of the tumor bearing mice suggesting that low-dose (0.75 mg/kg) CPA-7 may combat tumors through other mechanisms than growth inhibition. Indeed, we observed that whether in healthy or in tumor bearing mice, a trend toward increased body weight occurred in mice treated with 0.75 mg/kg CPA-7. Therefore, we have determined the optimal and the maximum dosage of CPA-7 in the RM-9 tumor model.

It has become evident that the immune surveillance plays a pivotal role in controlling cancerous growth. Some chemotherapeutic drugs not only directly inhibit proliferation of tumor cells but also influence anticancer immunity [26]. Platinum-based chemicals, either alone or in combination with other agents, serve as direct tumor inhibitors as well as immune modulators [22]. For example, oxaliplatin kills tumor cells directly but it also initiates immunosuppressive pathways, resulting in decreased activation of T cells by human plasmacytoid dendritic cells (pDCs) [27]. These unfavorable effects of platinum compounds may be related to the doses employed for treatment, because low-dose cisplatin combined with other modalities such as suicide gene therapy improves tumor clearance by increasing the number of tumor specific CD8+ T cells [28]. Our results clearly showed a significant elevation in CD4+ and CD8+ cytotoxic T lymphocytes (CTL) and a reduction in CD4 + CD25 + FOXP3+ Treg after treatment of mice with CPA-7. Considering the Treg inhibiting function of CPA-7, the increase of CD4+ population suggests that treatment with CPA-7 efficiently promotes the anti antigen activity of the immune system. Furthermore, down-regulation of Treg cells indicates that in addition to immunostimulation, CPA-7 may depress immunosuppressive pathways. Altogether, our results demonstrate that CPA-7 treatment is efficient in remodeling both immunostimulation and immunosuppression processes, conferring an enhanced immune activity against tumor recurrence and distant metastasis. This notion is evidenced by the significant low recurrence rate of the rechallenged tumors in this study, supporting CPA-7 as a potential synergistic pre-surgical or neo-adjuvant therapy for PC.

Our in vitro and in vivo results clearly demonstrated that the anti-tumor effects of CPA-7 are closely correlated with signal transducer and activator of transcription 3 (STAT3). STAT3 is a well known transcription factor that plays key roles not only in tumor development and tumor metastasis, but also in immune surveillance and immune response [11, 14]. Therefore inhibiting or demolishing the expression of active STAT3 is of great interest for both directly anti tumor treatment and tumor specific immunotherapy [11, 29, 30]. STAT3 promotes tumorigenesis by regulating the expression of various target genes, including cell-cycle regulators, angiogenic factors and anti-apoptosis genes [14, 31, 32]. Retrospective studies have established that STAT3 expression or phospho-STAT3 (pSTAT3 or activated STAT3) are poor prognostic markers for many cancers including PC [33]. Inhibition of STAT3 activity limited PC cell motility, possibly via transcriptive regulation of NKX3.1 and KLF4 gene expression by directly binding to both promoters [16, 34]. In our study, CPA-7 treatment reduced the phosphorylation of STAT3 resulting in a significant retardation of tumor progression, which underscores the pivotal role of STAT3 in PC treatment.

Among the immunosuppressive components that hinder the ability of the immune system to mount a sufficient immune response, STAT3 has been shown to function in the junction of several oncogenic and inflammatory pathways [35]. STAT3 is known to be activated by several immunosuppressive cytokines commonly found in the tumor microenvironment, such as IL-10, which, in turn, further promotes the expression of these cytokines through a positive feedback loop. Furthermore, STAT3 inhibits the expression of pro-inflammatory cytokines, such as IL-12, and has been demonstrated to employ infiltrating immune cells as a means of evading innate and adaptive immunity [36]. In this study, we found that the phosphorylation level of STAT3 strikingly decreased when the treatment of CPA-7 was adopted. The depletion of pSTAT3, in turn, led to a significant down-regulation of its transcriptional activity. In addition to the in vitro cultured cells, CPA-7 also inhibited the level of pSTAT3 in established tumor tissue. pSTAT3 expression was particularly inhibited in the tumor infiltrating region by CPA-7 treatment. This inhibition by CPA-7 may lead to the down regulation of FOXP3 and then account for the decreasing number of Treg cells in the immune system, because activated STAT3 induces expression of FOXP3, an essential transcription factor for Treg expansion [37]. In addition, our study further demonstrated that the population of CD8+ cytotoxic T cells was significantly expended after CPA-7 treatment, in support of the recent studies that STAT3 inhibitors abrogate immunosuppressive effects in PC patient-derived MDSCs on CD8^+^ T cells and increase the ratio of tumor-infiltrating CD8+ T cells to regulatory T cells, resulting in CD8+ T cell dependent regression of cancer [9, 38].

## Conclusion

In summary, our in vitro and in vivo studies demonstrate that CPA-7 is a potential novel therapeutic for treatment of prostate cancer, leading to decreased tumor burden and increased survival. Administration of CPA-7 effectively inhibited the level of pSTAT3 in vivo. pSTAT3 depletion in the tumor tissue and in the spleen and TDLN possibly accounted for reduction of tumor growth, decrease of Treg and increase of CTL in the immune system. These results indicate that CPA-7, in addition to promoting immunostimulation, concomitantly has an impact on immunosuppression. These beneficial effects of CPA-7 may contribute to the resultant prolonged survival of mice with prostate cell induced tumors. It is clear that comprehensive refinements and innovations in cancer treatment strategies are needed to eradicate the primary tumor while preventing tumor recurrence and distant metastasis. Tumor immunotherapy holds great promise, yet optimal strategies to balance the immunostimulative and immunosuppressive responses must be developed to produce clinically significant therapeutic benefits. Based on these concepts, in future studies we propose a series of experiments both to fully elucidate how STAT3 is involved in CPA-7 effects on the immune response and to maximize the tumor inhibition effects of CPA-7 for the clinical treatment of cancer.

## Abbreviations

ADT, androgen deprivation therapy; CTL, cytotoxic T lymphocytes; IHC, immunohistochemistry; PC, prostate cancer; pDCs, plasmacytoid dendritic cells; p-STAT3, phospho-STAT3; STAT3, signal transducer and activator of transcription 3; TDLNs, tumor draining lymph nodes; TH1, T helper 1; Tregs, regulatory T cells

## Additional file

Additional file 1: Figure S1. In vivo level of pSTAT3 after CPA-7 treatment. RM9 tumor tissues were collected 12 days after CPA-7 treatment. Immunohistochemistry was performed and pSTAT3 positive cells were stained in purple. Nuclei of pSTAT3 negative tumor cells were in blue. (TIF 1104 kb)
