# Supplementary figures and images for: CPA-7 influences immune profile and elicits anti-prostate cancer effects by inhibiting activated STAT3
Source: BMC Cancer. 2016 Jul 19;16:504. doi: 10.1186/s12885-016-2488-6 (PMC4952363; doi:10.1186/s12885-016-2488-6)

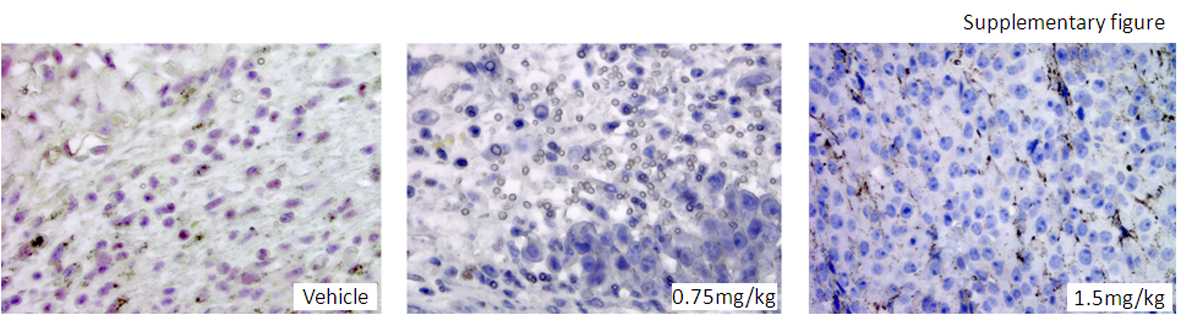

Supplement: Additional file 1: Figure S1. — In vivo level of pSTAT3 after CPA-7 treatment. RM9 tumor tissues were collected 12 days after CPA-7 treatment. Immunohistochemistry was performed and pSTAT3 positive cells were stained in purple. Nuclei of pSTAT3 negative tumor cells were in blue. (TIF 1104 kb) [file 12885_2016_2488_MOESM1_ESM.tif]
